# Umbilical cord-derived mesenchymal stromal cells attenuate radiation-induced neuron damage in vitro

**DOI:** 10.1186/s13287-026-04907-8

**Published:** 2026-01-22

**Authors:** Trang Thi Binh Pham, Kenshi Sei, Yuki Yamamoto, Takeo Mukai, Hiroyuki Akai, Tokiko Nagamura-Inoue

**Affiliations:** 1 Department of Cell Processing and Transfusion, The Institute of Medical Science, The University of Tokyo, 4-6-1 Shirokanedai, Minato-ku, Tokyo, 108-8639 Japan; 2 Human Life CORD Japan Inc, Nihonbashi Life Science Building 7, 1-9-10 Nihonbashi Horidomecho, Chuo-ku, Tokyo, 103-0012 Japan; 3 Division of Somatic Stem Cell Research, Center for Stem Cell Biology and Regenerative Medicine, The Institute of Medical Science, The University of Tokyo, 4-6-1 Shirokanedai, Minato-ku, Tokyo, 108-8639 Japan; 4 IMSUT CORD, The Institute of Medical Science, The University of Tokyo, 4-6-1 Shirokanedai, Minato-ku, Tokyo, 108-8639 Japan; 5 Department of Pediatrics, The University of Tokyo Hospital, Hongo, Bunkyo-Ku, Tokyo, 113-8655 Japan; 6 Department of Radiology, The Institute of Medical Science, The University of Tokyo, 4-6-1 Shirokanedai, Minato-ku, Tokyo, 108-8639 Japan

**Keywords:** Mesenchymal stromal cell, Umbilical cord, Neuroprotection, Radiation injury, Brain injury

## Abstract

Radiation-induced brain injury is caused by repeated radiation therapy for brain tumors and leukemia. Effective treatments for radiation-induced brain injury have not been developed. This study aimed to investigate the neuroprotective effects of umbilical cord-derived mesenchymal stromal cells (UC-MSCs) on irradiated neurons. We irradiated fetal mouse cortical neurons followed by coculture with UC-MSCs in vitro. Radiation significantly reduced the number of MAP2-positive mature and GAP43-positive immature neurons with a shortened neurite length, whereas coculture with UC-MSCs significantly restored the number and length of both MAP2-positive and GAP43-positive neurons. Irradiation induced apoptosis/necrosis in neurons significantly, while UC-MSCs prevented the neurons from apoptosis to necrosis. The incidence of reactive oxygen species (ROS) increased significantly in irradiated neurons compared to the control group, whereas it was significantly attenuated by the coculture of UC-MSCs. In conclusion, these results suggest that UC-MSCs have potential neuroprotective effects against radiation-induced brain injury by reducing oxidative stress.

## Introduction

Radiation therapy is a widely used and effective treatment for patients with malignant tumors, metastases, or leukemic infiltration in the central nervous system [1]. Cranial irradiation results in multifactorial encephalopathy due to differences in the radiosensitivity of various components of the brain parenchyma and vasculature [2]. Radiation-induced brain injury causes demyelination or selective oligodendrocyte dysfunction, focal brain apoptosis/necrosis and cognitive impairment. Late reactions include irreversible neurogenic damage with gliosis. Therefore, progression should be halted before irreversible damage can occur. Early-phase inflammation in radiation-induced brain injury is caused by the production of reactive oxygen species (ROS) that activate oxidative damage, microglial activation, neuronal death, blood–brain barrier (BBB) disruption and cerebral vascular damage [3, 4].

Mesenchymal stromal cells (MSCs) have immunomodulatory and tissue repair functions. MSCs can be obtained from several sources, including the bone marrow (BM), adipose tissue (AD), and umbilical cord (UC) [5, 6]. We previously reported that UC-MSCs were effective in curing neonatal cerebral palsy models because of their ability to suppress inflammation and secrete neurotrophic factors [7–9]. Compared with MSCs derived from bone marrow and adipose tissue, UC-MSCs can be obtained noninvasively from umbilical tissue, exhibit faster and greater proliferative potency, and greater migration potential to inflammatory blood cells [10]. Owing to these benefits, UC-MSCs are expected to be a source of radiation-induced brain injury.

This study investigated the neuroprotective effects of UC-MSCs on irradiated mouse cortical neurons.

## Materials and methods

The work has been reported in line with the ARRIVE guidelines 2.0.

### UC-MSC preparation

Ethics approval and consent to participate’ statement: Development of Novel Cell Therapy for Radiation Injury (No. 2019-80 and No. 2024 -05 since March, 19, 2020), including usage of human cell and animal experiments were obtained by the Ethics Committee of The Institute of Medical Science, The University of Tokyo (IMSUT). Human UC-MSCs were provided by the Umbilical Cord Blood (CB) and UC Bank of the Research Hospital, IMSUT (the bank name is IMSUT CORD https://plaza.umin.ac.jp/imsutcord/ ) after provision approval by IMSUT CORD steering committee (No. U0001_P0015). Ethics approval and consent to participate’ statement ‘IMSUT CORD project (No. C005/36-2)’were obtained by the IMSUT IRB according to the Declaration of Helsinki since 23, February, 2014.

UC-MSCs derived from three individual donors were used in this study. The procurement and processing of UC-MSCs have been described previously. Briefly, passage 1 (P1) cells were obtained by improved explant method in α-minimal essential medium (αMEM; Wako Pure Chemical Industries, Ltd., Japan) supplemented with 10% fetal bovine serum and antibiotics-antimycotics (Antibiotic-Antimycotic, 100X; Life Technologies, USA) at 37 °C with 5% CO_2_, collected by trypsin (TrypLE Select, Life Technologies, USA) and cryopreserved in Stemcell Banker (Zenoaq, Japan). The frozen-thawed P1 cells were then cultured until P4 cells were used for the experiments.

### Cortical neuron primary cultures

The animal experiments ‘Research on immune and regenerative medicine using UC and CB-derived cells (No. A21-68 approved on March 23, 2022) and ‘Research of UC and CB banking and development of their applications (No.2025IMS022 approved on 25, September, 2023)’ were approved by the intramural animal research review committee of the IMSUT. The experiment was conducted in animal center of IMSUT, in compliance with the Animal Centre’s regulations.

B6 albino mice (B6N-Tyrc-Brd/BrdCrCrl) were purchased from Charles River Laboratories International, Japan.

We carried out primary cultures of cortical neurons as follows. Briefly, we performed the mating of the ten paired mice, then, got the nine pregnant mice and estimated the birth date. On embryonic day 17, the fetuses were collected from three euthanized pregnant mice by inhaling CO_2_ without anesthesia, and the fetal brains were removed. The cortical tissues of fetal brains were dissected under a microscope and minced into small pieces. The cells were then dispersed using neuron dissociation solutions (Wako Pure Chemical Industries, Ltd., Japan) and filtered. Finally, the collected neurons were resuspended in neurobasal medium (GIBCO) supplemented with 2% B27 and plated onto Poly-d-Lysine Culture dishes (BioCoat™, Corning Inc. Japan). Neurons were cultured at 37 °C with 5% CO_2_ for 3–5 days, and half of the medium was replaced with fresh medium for 7 days.

### Irradiation and coculture with UC-MSCs

Mouse primary cortical neurons were irradiated with a single dose of 12 Gy [4] and immediately cocultured with UC-MSCs. The primary cortical neurons were cultured at 1.5 × 10^5^ cells /well at the bottom of a 24-well transwell chamber (Corning Inc.) equipped with a 3-µm filter membrane while UC-MSCs were plated in the upper chamber at 1.5 × 10^4^ cells/well overnight at 37 °C with 5% CO_2_ for 72 h [11, 12]. We compared 3 groups including in this study: no irradiated neurons (control group); irradiated neurons (Radiation); and irradiated neurons cocultured with UC-MSCs (UC-MSC coculture group).

### Immunocytochemical evaluation of cortical neurons

Cortical neurons were fixed with 4% paraformaldehyde for 20 min at room temperature. The primary antibodies used were MAP2; 1:1000 dilution; and GAP43; 1:200 dilution. Nuclei were counterstained with DAPI. A quantitative analysis of neurite length was performed on MAP2-positive mature neurons and GAP43-positive immature neurons by counting the fluorescent cells in 10 fields of the samples.

### Apoptosis assay

The apoptosis assay was performed according to the manufacturer’s instructions (PromoKine; PromoCell GmbH, Germany). The neurons were exposed to 5 µl of FITC-Annexin V and 5 µl of ethidium homodimer III. The samples were incubated with the solution for 15 min at room temperature and fluorescence was observed via FITC and Texas Red filter sets. Quantitative analysis was performed by the counting the fluorescent cells from 10 fields of the samples and shown in the ratio.

### ROS assay

Reactive oxygen species (ROS) levels were quantified via the DCFDA/H2DCFDA-Cellular ROS Assay Kit. A quantitative analysis of ROS-positive cells was performed by counting the fluorescent cells in 10 fields of the samples.

### Statistical analysis

The values are expressed as the means ± standard deviations (SDs) from three independent experiments. Differences between groups were analyzed with ‘R’ (https://cran.r-project.org/). Groups were compared via one-way analysis of variance (ANOVA), followed by Tukey’s test. P values of 0.05 or less were regarded as statistically significant.

## Results

### UC-MSCs restored the number and neurite length of irradiated neurons

**Fig. 1:**
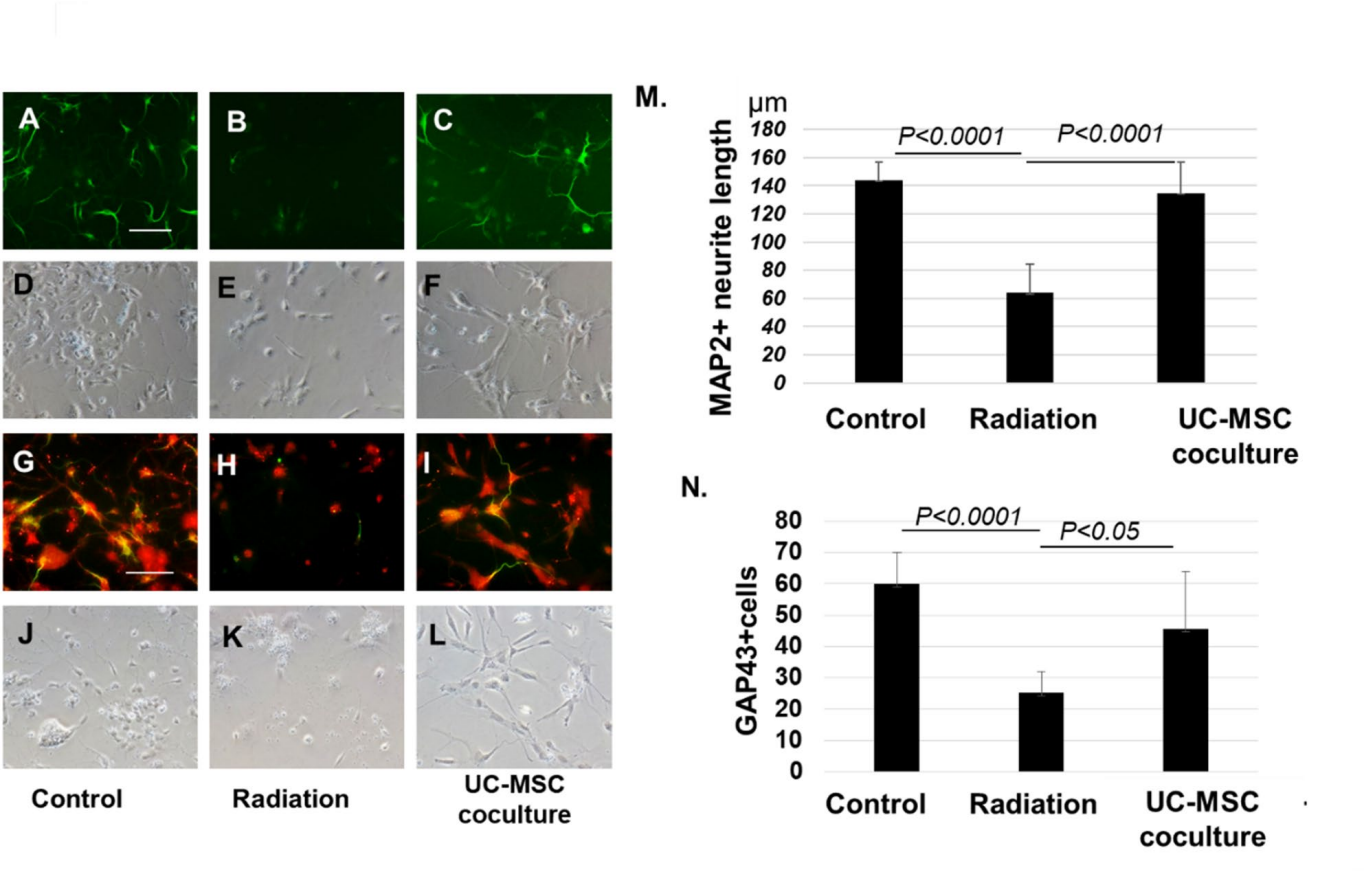
UC-MSCs restore irradiated injured fetal cortex neurons. Mouse fetal cortical neurons were irradiated and cocultured with or without UC-MSCs. Mature neurons are MAP2 positive (green fluorescence), proliferating immature neurons are GAP43 positive (red fluorescence), and nuclei are counterstained with DAPI (blue). **A**, **D**, **G**, and **J** Fetal cortex neurons without radiation (Control), **B**, **E**, **H**, and **K** Irradiated neurons (Radiation). **C**, **F**, **I**, and **L** Irradiated neurons cocultured with UC-MSCs (UC-MSC coculture). MAP2 staining in **A**–**C** and their morphologies in **D**–**F**, GAP43 staining in **G**–**I** and their morphologies in **J**–**L**. The representative fluorescent pictures are shown from three individual experiments. Quantitative study of neurite length in MAP2-positive mature neurons (**M**). Quantitative study of GAP43-positive immature neurons (**N**), Scale bar = 100 μm. UC-MSCs, umbilical cord-derived mesenchymal stromal cells

We investigated the influence of UC-MSCs on irradiated mouse cortical neurons on the basis of neuron number and neurite length. After 72 h of irradiation, the number and neurite length of MAP2-positive mature neurons (green fluorescence) and GAP43-positive immature neurons (red fluorescence) decreased or disappeared in the neurons after irradiation (radiation group) compared with those in nonirradiated neurons (control group). In contrast, coculture with UC-MSCs (UC-MSC coculture group) significantly restored the number and length of MAP2-positive neurons (*P* < 0.0001, Fig. 1A-F, and 1M). In addition, the number and length of GAP43-positive immature proliferating neurons were lower in the radiation group than in the control group (*P* < 0.0001, Fig. 1G–L, N). In contrast, coculture of UC-MSCs with irradiated neurons significantly restored the number of GAP43-positive immature neurons (*P* < 0.05, Fig. 1N).

### Influence of UC-MSCs on the apoptosis/necrosis ratio of irradiated neurons

**Fig. 2:**
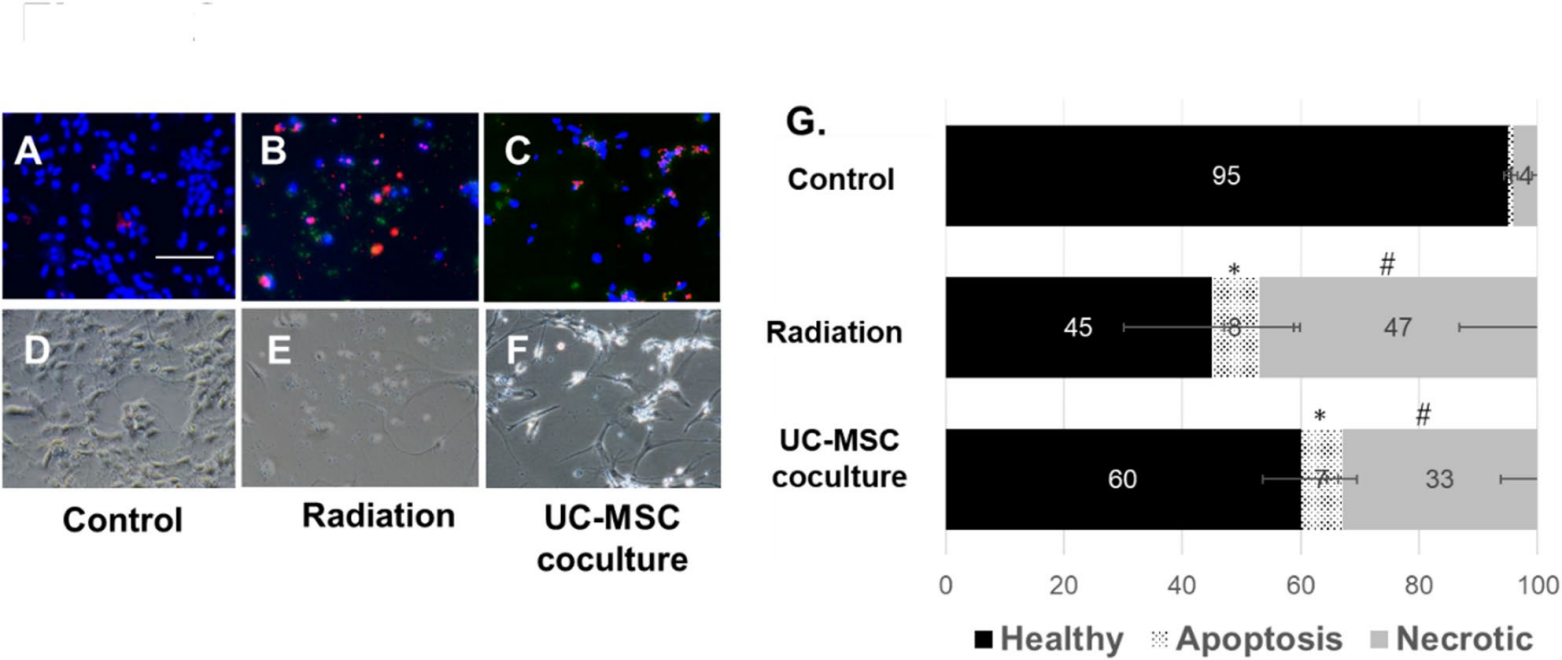
UC-MSCs protect against neuron necrosis after irradiation. Radiation-injured fetal cortical neurons were cocultured with or without UC-MSCs. Apoptotic neurons are Annexin V-positive (green), and necrotic neurons are ethidium homodimer III-positive (red) in **A**–**C** with DAPI staining (blue), with their morphologies shown in **D**–**F**. **G** Quantitative percentages of living, apoptotic, and necrotic cells in the control group; irradiated neurons (Radiation); and irradiated neurons with UC-MSCs (UC-MSC coculture). Scale bar = 100 μm. * *P* < 0.05 (Control versus Radiation and Control versus UC-MSC coculture group, Not significant between Radiation and UC-MSC coculture group) # *P* < 0.01 (Control versus Radiation versus UC-MSC coculture)

Next, we assessed the neuroprotective effect of MSCs on the apoptosis of irradiated neurons and necrosis. Quantitative analysis revealed that the ratio of apoptotic (green fluorescence) and necrotic (red fluorescence) cells in all neurons was significantly higher in the radiation group and UC-MSC coculture group than in the control group (Fig. 2A–F). But the ratio of necrotic cells was restored by coculture with UC-MSCs compared with those in the radiation group, significantly (Fig. 2G), although the ratio of apoptosis was not attenuated by the UC-MSC coculture.

### UC-MSCs reduced ROS in irradiated neurons

**Fig. 3:**
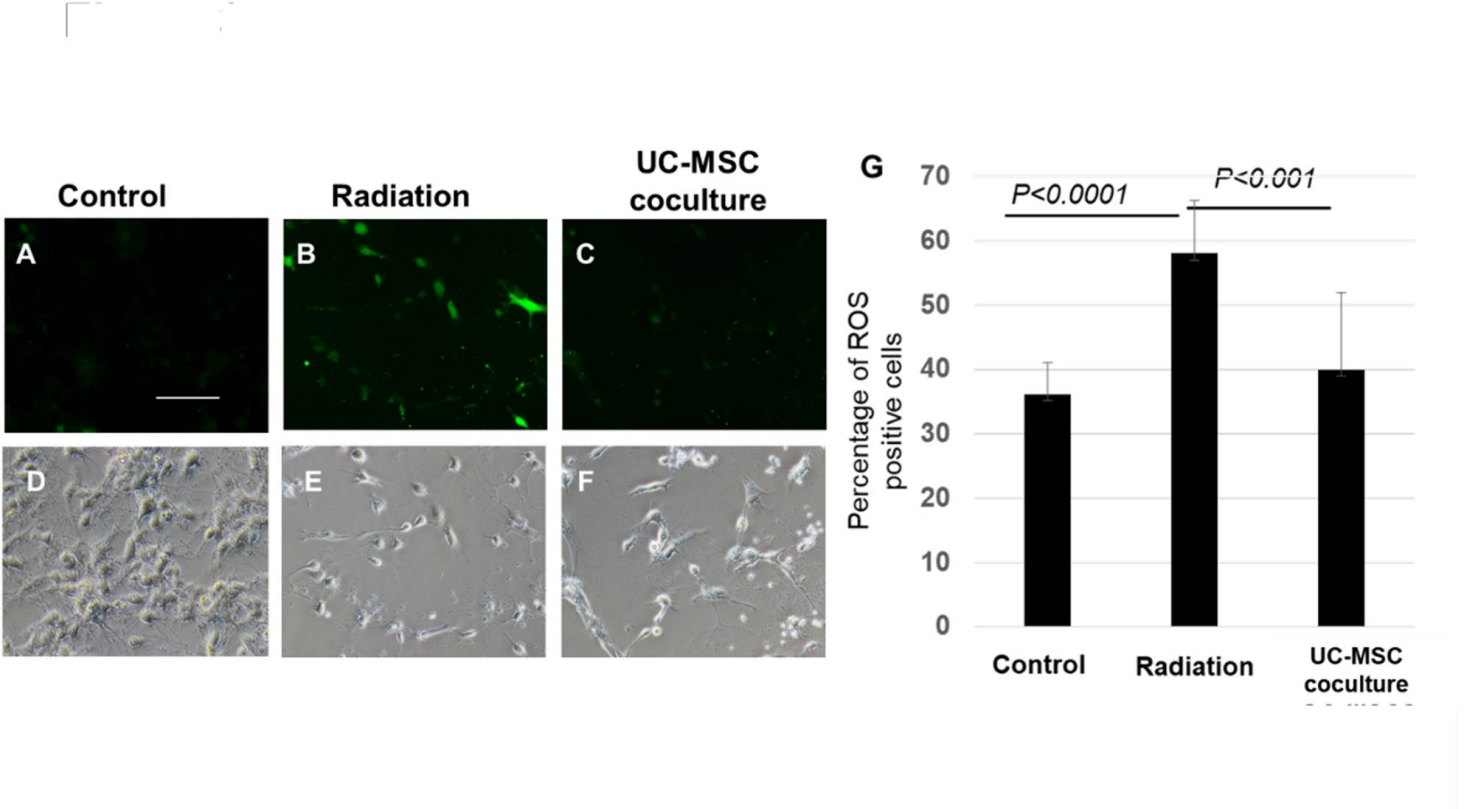
UC-MSCs reduce reactive oxygen species (ROS) in irradiated cortical neuronsReactive oxygen species (ROS) were measured in radiation-injured fetal cortex neurons cocultured with or without UC-MSCs via the ROS assay kit described in the Materials and methods. ROS-positive (green fluorescence) cells in the neurons are shown in green in A-C with morphology in D-F. The percentage of total ROS-positive cells is shown in G. Scale bar = 100 µm. P<0.0001 (Control versus Radiation). P<0.001 (Radiation versus UC-MSC coculture)

We evaluated ROS activity to assess the mechanism of neuronal damage following irradiation. We calculated the percentage of ROS-positive (green fluorescence) cells per total number of neurons in each of the 10 fields (Fig. 3A–F). We demonstrated that the incidence of ROS-positive 

cells was significantly greater in the radiation group than in the control group (*p* < 0.0001), whereas it was significantly lower in irradiated neurons cocultured with UC-MSCs than in those in the radiation group (*p* < 0.001) (Fig. 3G). The incidence of ROS-positive cells was 36 ± 4.85% in the control group, 58 ± 8.25% in the radiation group, and 39 ± 12.11% in the UC-MSC coculture group.

## Discussion

Radiation injury represents a growing concern regarding the long-term adverse effects of ionizing radiation on nervous tissues for treating malignancies in the brain. However, the real world remains under the threat of nuclear weapons or accidents in nuclear power industries, such as those of Chernobyl and Fukushima (Japan) [13, 14]. Therefore, there is a great need to better understand radiation sickness. Here, we established a radiation-induced brain injury model in vitro and cocultured with UC-MSCs; in doing so, we proved that UC-MSCs have neuroprotective effects after the irradiation of neurons. The impact of pulse radiation on a single cell may immediately affect vital pathways that control redox balance, resulting in the apoptosis or necrosis of neurons accompanied by the release of ROS.

UC-MSCs represent a promising source for treating brain injury, as previously reported [8, 15]. In addition, their effects have been demonstrated in clinical trials [16] in the context of various diseases, including acute graft-versus-host disease, cerebral palsy, ischemic brain injury, heart disease, and collagen disease. UC-MSCs may be feasible for clinical use now.

In our in vitro model, direct irradiation of fetal neurons induced a shortened neurite length and decreased the number of immature neurons expressing GAP43, which were restored by coculture with UC-MSCs. These results mimic the neuroprotective potency of UC-MSCs in the context of neurogenic damage resulting from oxygen‒glucose deprivation [9]. The ratio of apoptotic and necrotic cells significantly increased following irradiation, and the ratio of necrotic cells was restored by coculture with UC-MSCs, significantly, although the ratio of apoptosis was not attenuated by the UC-MSC coculture. The coculture with UC-MSCs might prevent the proceeding of the neurons from apoptosis to necrosis. Wang et al. reported whole brain irradiation in C57BL/6 mice induced neuronal degeneration and necrosis while those were less in UC-MSCs administration group [17].

The mechanisms of radiation injury have been studied with respect to redox systems. Radiation injury causes neurons to release large amounts of ROS via dysregulated mitochondria [2, 18]. The radiation-induced imbalance of the mitochondrial redox machinery represents the major consumer of oxygen in aerobes and is the major source of metabolically produced ROS in most cells [2]. Therefore, the suppression of ROS is a critical target for restoring the injury of irradiated neurons. Alhazzani et al. demonstrated that ischemic-stressed neuronal cells were rescued from death induced by the control of [Ca(2+)]i and ROS, followed by increased expression of anti-inflammatory cytokines such as IL-10, TGF-β, and SOD1. Indoleamine 2,3-dioxygenase 1 (IDO-1) functions as an antioxidant via the kynurenine pathway in tryptophan metabolism and is induced to be secreted from UC-MSCs under inflammatory conditions.

In conclusion, irradiation induced a decrease in mature neurons, accompanied by a decrease in neurite length. The neuronal damage caused by irradiation was reversed by coculturing with UC-MSCs, through reduced ROS levels and necrosis, although apoptosis was not reduced by coculture of UC-MSCs. UC-MSCs may be effective and feasible treatments for radiation-induced brain injury.

## Data Availability

The datasets used and/or analysed in the current study are available from the corresponding author on reasonable request. All experimental data are included in the manuscript.

## References

[1] Parihar VK, Limoli CL. Cranial irradiation compromises neuronal architecture in the hippocampus. Proc Natl Acad Sci U S A. 2013;110(31):12822–7. 23858442 10.1073/pnas.1307301110 PMC3732939

[2] Gorbunov NV, Kiang JG. Brain damage and patterns of neurovascular disorder after ionizing irradiation. Complications in radiotherapy and radiation combined injury. Radiat Res. 2021;196(1):1–16. 33979447 10.1667/RADE-20-00147.1 PMC8297540

[3] Xu L, et al. Exposure to X-rays causes Depression-like behaviors in mice via HMGB1-mediated pyroptosis. Neuroscience. 2022;481:99–110. 34800578 10.1016/j.neuroscience.2021.11.023

[4] Yang L, et al. Pathophysiological responses in rat and mouse models of radiation-induced brain injury. Mol Neurobiol. 2017;54(2):1022–32. 26797684 10.1007/s12035-015-9628-x PMC5310567

[5] Gnecchi M, Melo LG. Bone marrow-derived mesenchymal stem cells: isolation, expansion, characterization, viral transduction, and production of conditioned medium. Methods Mol Biol. 2009;482:281–94. 19089363 10.1007/978-1-59745-060-7_18

[6] Gruber HE, et al. Human adipose-derived mesenchymal stem cells: direction to a phenotype sharing similarities with the disc, gene expression profiling, and coculture with human annulus cells. Tissue Eng Part A. 2010;16(9):2843–60. 20408770 10.1089/ten.TEA.2009.0709

[7] Mukai T, et al. Neurosphere formation enhances the neurogenic differentiation potential and migratory ability of umbilical cord-mesenchymal stromal cells. Cytotherapy. 2016;18(2):229–41. 26794714 10.1016/j.jcyt.2015.10.012

[8] Mukai T, et al. Intravenous injection of umbilical cord-derived mesenchymal stromal cells attenuates reactive gliosis and hypomyelination in a neonatal intraventricular hemorrhage model. Neuroscience. 2017;355:175–87. 28504197 10.1016/j.neuroscience.2017.05.006

[9] Mukai T, Tojo A, Nagamura-Inoue T. Umbilical cord-derived mesenchymal stromal cells contribute to neuroprotection in neonatal cortical neurons damaged by oxygen-glucose deprivation. Front Neurol. 2018;9:466. 29963009 10.3389/fneur.2018.00466 PMC6013549

[10] Hori A, et al. Superior migration ability of umbilical cord-derived mesenchymal stromal cells (MSCs) toward activated lymphocytes in comparison with those of bone marrow and adipose-derived MSCs. Front Cell Dev Biol. 2024;12:1329218. 38529405 10.3389/fcell.2024.1329218 PMC10961348

[11] Alhazzani A, et al. Mesenchymal stem cells (MSCs) coculture protects [Ca2+]i orchestrated oxidant mediated damage in differentiated neurons in vitro. Cells. 2018;7(12):250. 30563298 10.3390/cells7120250 PMC6315478

[12] Mariana A, et al. Mesenchymal stem cells and cell-derived extracellular vesicles protect hippocampal neurons from oxidative stress and synapse damage induced by amyloid-β oligomers. J Biol Chem. 2018;293(6):1957–75. 29284679 10.1074/jbc.M117.807180 PMC5808759

[13] Nagayama H, et al. Transient hematopoietic stem cell rescue using umbilical cord blood for a lethally irradiated nuclear accident victim. Bone Marrow Transpl. 2002;29(3):197–204. 10.1038/sj.bmt.1703356 11859391

[14] Nagayama H, et al. Severe immune dysfunction after lethal neutron irradiation in a JCO nuclear facility accident victim. Int J Hematol. 2002;76(2):157–64. 12215015 10.1007/BF02982579

[15] Cheng Q, et al. Human umbilical cord mesenchymal stem cells protect against ischemic brain injury in mouse by regulating peripheral immunoinflammation. Brain Res. 2015;1594:293–304. 25449888 10.1016/j.brainres.2014.10.065

[16] Forostyak S, Jendelova P, Sykova E. The role of mesenchymal stromal cells in spinal cord injury, regenerative medicine and possible clinical applications. Biochimie. 2013;95(12):2257–70. 23994163 10.1016/j.biochi.2013.08.004

[17] Wang G, et al. Neuroprotective effects of umbilical cord-derived mesenchymal stem cells on radiation-induced brain injury in mice. Ann Clin Lab Sci. 2020;50(1):57–64. 32161012

[18] Kobashigawa S, Suzuki K, Yamashita S. Ionizing radiation accelerates Drp1-dependent mitochondrial fission, which involves delayed mitochondrial reactive oxygen species production in normal human fibroblast-like cells. Biochem Biophys Res Commun. 2011;414(4):795–800. 22005465 10.1016/j.bbrc.2011.10.006

